# Sacroiliac versus transiliac–transsacral screw osteosynthesis in osteoporotic pelvic fractures: a biomechanical comparison

**DOI:** 10.1007/s00068-023-02341-6

**Published:** 2023-08-03

**Authors:** Raffael Cintean, Cornelius Fritzsche, Ivan Zderic, Boyko Gueorguiev-Rüegg, Florian Gebhard, Konrad Schütze

**Affiliations:** 1 Department of Trauma-, Hand-, and Reconstructive Surgery, Ulm University, Albert-Einstein-Allee 23, 89081 Ulm, Germany; 2 Department of Handsurgery, Upper Extremities and Foot Surgery, Krankenhaus Waldfriede, Argentinische Allee 40, 14163 Berlin, Germany; 3 AO Research Institute (ARI), Clavandelerstrasse 8, 7270 Davos Platz, Switzerland

**Keywords:** Osteoporotic pelvic fracture, Biomechanical, Sacroiliac screw, Fragility fracture

## Abstract

**Introduction:**

Pelvic fractures were often associated with high-energy trauma in young patients, but data show a significant increase in osteoporotic pelvic fractures in old age due to the progressive demographic change. There is an ongoing discussion about the best fixation techniques, which are ranging from lumbopelvic fixation to sacral bars or long transiliac–transsacral (TITS) screws. This study analyzes TITS screw osteosynthesis and sacroiliac screw osteosynthesis (SI), according to biomechanical criteria of fracture stability in osteoporotic human pelvic cadavers ex vivo.

**Methods:**

Ten osteoporotic cadaveric pelvises were randomized into two groups of 5 pelvises each. An FFP-IIc fracture was initially placed unilaterally and subsequently surgically treated with a navigated SI screw or a TITS screw. The fractured side was loaded in a one-leg stance test setup until failure. Interfragmentary movements were assessed by means of optical motion tracking.

**Results:**

No significant difference in axial stiffness were found between the SI and the TITS screws (21.2 ± 4.9 N and 18.4 ± 4.1 N, *p* = 0.662). However, there was a significantly higher stability of the fracture treatment in the cohort with TITS-screws for gap angle, flexion, vertical movement and overall stability. The most significant difference in the cycle interval was between 6.000 and 10.000 for the gap angle (1.62 ± 0.25° versus 4.60 ± 0.65°, *p* = 0.0001), for flexion (4.15 ± 0.39 mm versus 7.60 ± 0.81 mm, *p* = 0.0016), interval 11.000–15.000 for vertical shear movement (7.34 ± 0.51 mm versus 13.99 ± 0.97 mm, *p* < 0.0001) and total displacement (8.28 ± 0.66 mm versus 15.53 ± 1.07 mm, *p* < 0.0001) for the TITS and the SI screws.

**Conclusions:**

The results of this biomechanical study suggest a clear trend towards greater fracture stability of the TITS screw with significantly reduced interfragmentary movement. The application of a TITS screw for the treatment of the osteoporotic pelvic ring fracture may be prioritized to ensure the best possible patient care.

## Introduction

The incidence of pelvic ring fracture, measured as a proportion of all fractures, is low, amounting to 3–8% [1–3]. However, entailing high mortality and morbidity, it poses enormous challenges to both the medical and the health care system. Pelvic fractures were often associated with high-energy trauma in young patients, but data show a significant increase in osteoporotic pelvic fractures in old age due the progressive demographic change [4]. To treat these fractures adequately, appropriate therapy algorithms are required. For this purpose, medicine applied existing treatment strategies used for pelvic ring fractures in the young patient, despite significant differences in terms of accident mechanism, fracture morphology, bone quality, and the patient itself [3, 5, 6]. In the geriatric patient, osteosynthesis must not only achieve sufficient fracture stabilization of the osteoporotic pelvic bone ensuring immediate full weight-bearing and allowing full mobilization, but it must also provide an acceptable perioperative risk considering the potentially accompanying multiple comorbidities [7, 8]. 

An evaluation of the German pelvic register from 1991 to 2003 shows a significant increase in the number of surgically treated cases [9]. However, the type of best surgical therapy continues to be discussed. There is an ongoing discussion about the best fixation techniques, which are ranging from lumbopelvic fixation to sacral bars or long transiliac–transsacral (TITS) screws [10]. Although minimally invasive surgical techniques have been established, literature on biomechanical studies evaluating the fracture stability using these techniques remains scarce up to date [11, 12]. These fixation techniques are well-established in young non-osteoporotic patients. Literature shows also promising long-term results in the treatment of fragility fractures of the pelvis but only in small study populations [7, 13, 14].

Various studies with bone models or embalmed pelvic cadavers can be found in the literature [13, 15–17]. Studies on fresh frozen human osteoporotic bones, however, are searched for in vain. This study biomechanically analyzes and compares the most used minimally invasive osteosynthesis procedures, SI and TITS screw osteosynthesis, in an osteoporotic human pelvic cadaveric model.

## Materials and methods

### Specimens and preparation

Institutional and prior ethical committee approval was obtained prior to the study. All pelvises were from donors who bequeathed their corpses to Science Care (Phoenix, AZ, USA) for use in medical science during their lifetime. Written consent by the patients before the decease or authorized family members is available. All experiments were carried out under the relevant guidelines and regulations of the local institution.

Ten fresh frozen (− 20°) cadaver female pelves from donors aged 87 ± 5 years (mean ± standard deviation, SD) (range 80–94 years) with intact sacroiliac, sacrospinous, sacrotuberous and symphyseal ligaments including the attached fifth lumbar vertebra were considered for biomechanical testing. To provide a consistent result of the biomechanical study, any specimens presenting pre-existent deformations, fractures, neoplasms or degenerative changes in the sacroiliac joint were excluded from the study. Bone mineral density (BMD) was measured before acquisition for each specimen with dual-emission X-ray absorptiometry at the level of S1. Based on BMD, the specimens were randomized into two non-paired groups for instrumentation using either an SI screw or a TITS screw, such that both anatomical sites of each pelvis received the same fixation technique, however, in sequential order comprising preparation, instrumentation and testing of one unilateral anatomical site first. Mean values of both cohorts are shown in Table 1. The Shapiro–Wilk test showed no difference between the groups regarding the distribution.

**Table 1:** Mean values of both cohorts

Cohort	Number (*n*)	Age (years)	BMI (kg/m^2^)	*T*-score
TITS	5	85.6 ± 3.8	15.7 ± 2.5	− 2.21 ± 0.61
SI	5	85.9 ± 5.8	16.9 ± 3.5	− 2.59 ± 0.67

*TITS* transiliac–transsacral, *SI* sacroiliacal

Prior to preparation and biomechanical testing, the specimens were thawed at room temperature for 24 h. Tissue dissection of skin and muscles was done carefully and all ligaments were preserved. After dissection, a fragility fracture of the pelvis (FFP) type IIc was simulated by means of osteotomies using a 1.47 mm oscillating surgical saw [18]. Therefore, the sacrum was cut between the SI-Joint and the neuroforamina in the sacral ala creating a vertical paraforaminal sacrum fracture in zone 1 after Denis classification [19]. Subsequently, the superior and inferior pubic rami were cut approximately 3 cm laterally from the symphysis to discontinue the force transmission to the contralateral hemipelvis site through the anterior pelvic ring during load application.

Under normal operating conditions, the specimens were reduced and treated with either a navigated SI-screw or TITS using a hybrid operating room which consists of a fixed robotic 3D flatpanel detector (Artis zeego, Siemens Healthineers, Germany) and a navigation system (BrainLab Curve, BrainLab, Germany). This ensured an optimal screw pathway for each pelvis. The fractured sacrum was anatomically reduced together with the superior and inferior pubic rami. Fully threaded self-tapping stainless steel 7.3 mm cannulated screws (DePuy Synthes, Zuchwil, Switzerland) were used with a standard washer for fracture fixation. Screw length was chosen specimen individually and according to the group assignment. Whereas for the SI screw fixation technique, care was taken that the screw tip did not cross the midline, for the TITS fixation technique, the screw tip was aimed to perforate all six cortices and extrude from the contralateral ilium. To create a machine fixation point, the L5-vertebrae was embedded in polymethylmethacrylate (PMMA, SCS-Beracryl D-28, Swiss Composite, Jägenstorf, Switzerland) cylinder, such that with the former oriented vertically, the pelvic tilt measured 45°. The fixation was enhanced with 5.0 mm screws. Finally, optical markers were attached to each side of the sacrum and superior ramus fracture, and to the ilium for motion tracking.

Due to the deterioration of the fresh frozen pelvis caused by thawing, two tests in the SI screw group and one test in the TITS screw group could not be completed and were excluded from the final evaluation.

### Biomechanical testing

**Fig. 1:**
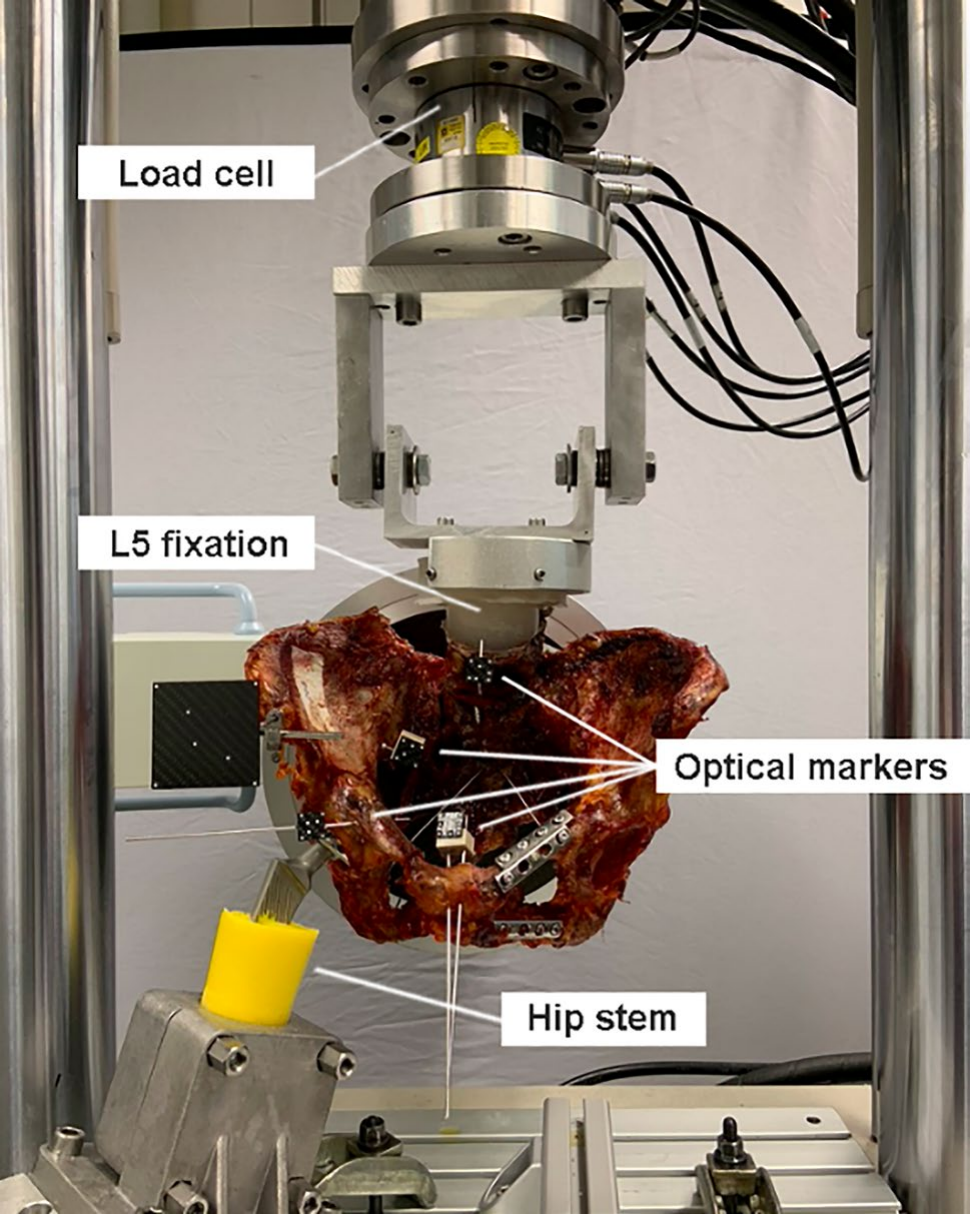
Test setup with specimen mounted for biomechanical testing

Biomechanical testing was performed on a servo hydraulic material testing system (MTS 858 Bionix, MTS Systems Corp., Eden Prairie, USA) equipped with a 4 kN load cell. Specimens were mounted to the machine in simulated upright standing position by fixing the L5 embedding to the machine transducer via an interconnected hinge joint, allowing for bending moment compensation in flexion–extension. One-leg stance was simulated by transferring the forces generated by the transducer, through the tested hemi-pelvic site to the ipsilateral acetabulum, which was seated on a hip stem with attached hemiarthroplasty component. The latter was firmly constrained to the machine base (Fig. 1).

The loading protocol commenced with an initial quasi-static ramp from 20 N preload to 100 at a rate of 8 N/s, followed by cyclic loading at 3 Hz under a physiological loading profile [20]. Whereas the valley load of 20 N was held constant, the peak load, starting at 100 N, was progressively increased at 0.006 N/cycle until the transducer reached 70 mm displacement with respect to its initial position prior to test start. The latter was found sufficient in a priori pilot tests to provoke failure in the bone-implant constructs and to retrospectively analyze the data for clinically relevant parameters.

After failure, whereas the dislocated fragments of tested anatomical side were anatomically re-aligned and re-fixed at the sacrum by means of 3 SI-Screws, the rami were re-fixed by plating. Subsequently, the procedure of fracture creation, stabilization, and testing was repeated on the contralateral side.

### Data acquisition and analysis

Machine data in terms of axial displacement (mm) and load (N) were acquired at 128 Hz. Based on them, the initial construct stiffness was calculated from the ascending load–displacement curve of the initial quasi-static ramp within the load range 50–90 N.

Two optical cameras (Aramis SRX, Carl Zeiss GOM Metrology GmbH, Braunschweig, Germany), operating at 12 Megapixel, continuously recorded the three-dimensional positions of the attached markers at 20 Hz throughout the tests. Based on these, the relative movements between the medial and lateral aspects of the sacrum fracture were analyzed with respect to the marker positions at the beginning of the cyclic test, by means of calculation of the following parameters:

(i) gap angle—angular displacement describing the combined fracture gap opening in frontal and transverse plane;
(ii) flexion—angular displacement describing relative fragment rotation in sagittal plane;
(iii) total displacement—magnitude of the translational displacement of the fracture aspect lying most anteriorly in the fracture gap.

Finally, to visually evaluate the gradual decay of the bone-implant constructs, antero-posterior X-rays were taken with a C-arm (Arcadis Varic, Siemens Healthineers, Erlangen, Germany) at test start, and then intermittently every 500 cycles in peak load condition. For that purpose, the cyclic test was interrupted for 2s in the corresponding load magnitude.

**Fig. 2:**
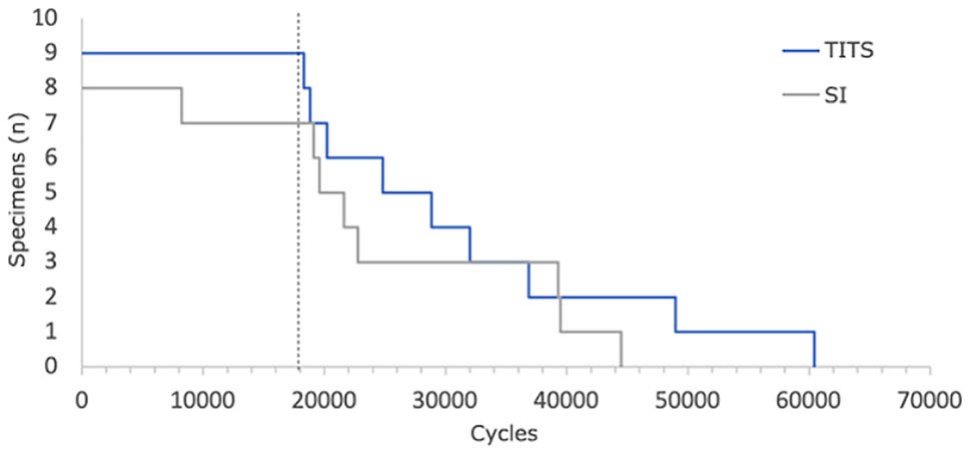
Implant failure (TITS: transiliac–transsacral, SI: sacroiliacal)

The outcome measures of these parameters were evaluated in peak loading conditions over the first 18,000 cycles in equidistant intervals of 1000 cycles. This range was chosen, because beyond it, the first specimens dropped out due to their failure (Fig. 2).

Statistical analysis was performed with SPSS Statistics (V21.0, IBM, Armonk, NY) and Graphpad (GraphPad Software, La Jolla California USA). Cohorts were tested with the Mann–Whitney *U* test for independent samples with not normally distributed data. Data pooling was done with time steps from 0 to 5000, 6000 to 10,000 as well as 11,000 to 15,000 cycles. Survival was defined by the failure of osteosynthesis or the maximum dislocation of the fragments, so that a measurement based on the optical markers can no longer be performed. The median survival rate of the implant was calculated using the Kaplan–Meier estimates and tested between the cohorts using a log-rank test. The level of significance was set to 0.05 for all statistical tests.

## Results

### Axial stiffness

The axial stiffness of the preparations was calculated via the compressive load of the servo-hydraulic testing machine. In order to be able to exclude an initial alignment, only a load interval of 50–90 N was included from the ramp test. This showed a higher stiffness in the long screw group without statistical significance (*p* = 0.662).

### Fracture displacement

**Fig. 3:**
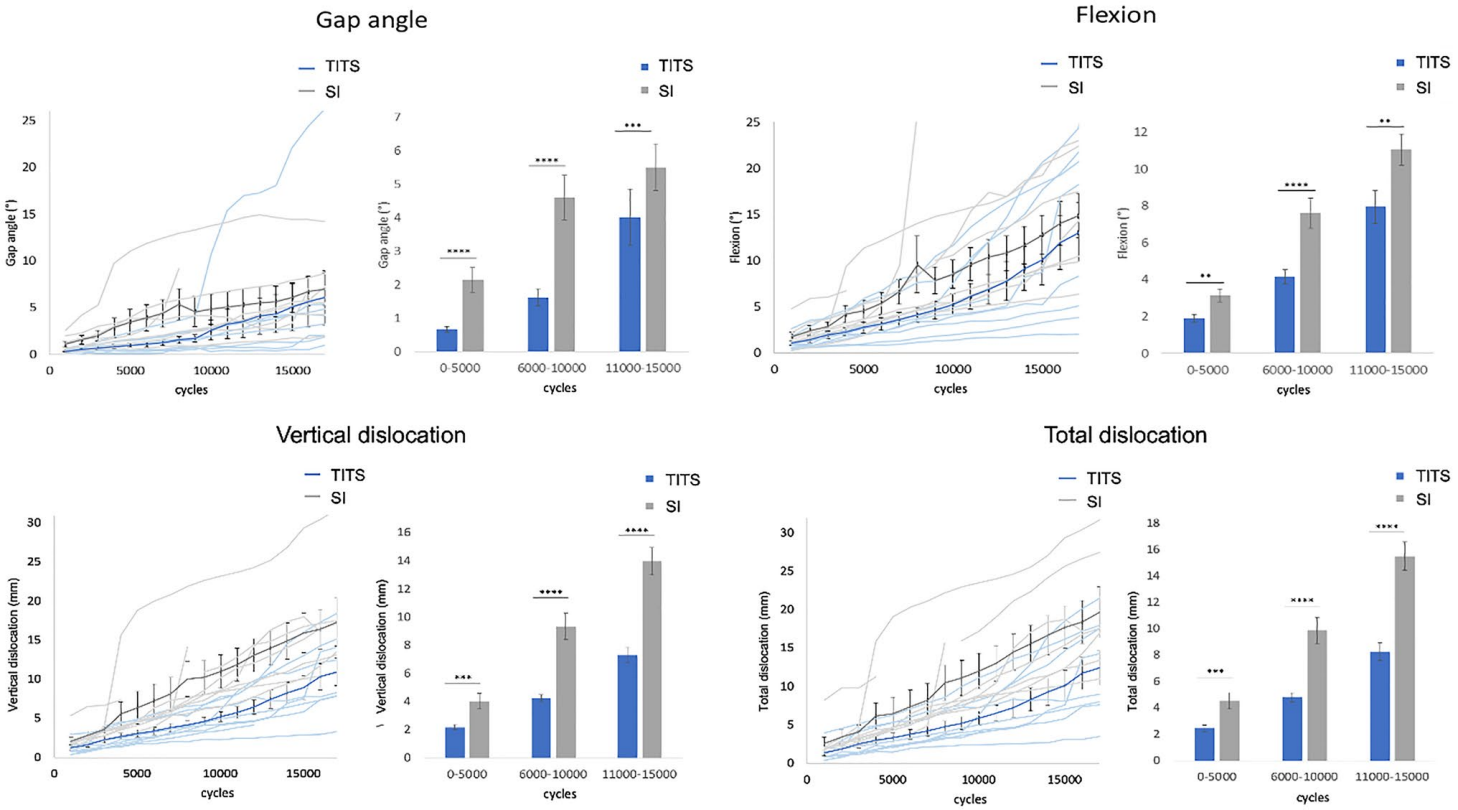
Gap angle, flexion, vertical dislocation and total dislocation. Thin blue lines for every specimen of the TITS group and grey thin lines for SI group. Thick lines show the mean and standard deviation for each group. Bar charts show mean values with standard deviation for the groups every 5000 cycles. **p < 0.01, ***p < 0.001, ****p < 0.0001

Outcome measures for the four parameters measured over the first 180,000 cycles are shown in Fig. 3 for intervals every 1000 and 5000 cycles, as well as for each group separately.

The mean gap angle was significantly higher in the SI group for all three intervals with the highest difference between 6000 and 10,000 cycles with 2.9° (*p* < 0.0001).

### Flexion

The mean flexion in the plane of fracture was significantly higher in the SI group for all cyclic intervals (*p* < 0.01). After initial dislocation, the flexion was mainly limited through the ligaments. After ligament failure, flexion for both groups approximated. The highest significance was observed between 6000 and 10,000 cycles (*p* < 0.0001).

### Vertical dislocation

The mean vertical dislocation was significantly higher in the SI group (*p* < 0.001). Dislocation increased with rising number of cycles. The difference between the groups was the highest between 11,000 and 15,000 cycles with 5.1 mm. The highest significances were observed between 6000 and 10,000 cycles (*p* < 0.0001).

### Total dislocation

The mean total dislocation was significantly higher in the SI group with an earlier and higher dislocation (*p* < 0.001). The highest total dislocation difference between the groups was seen between 11,000 and 15,000 cycles with 5.0 mm (*p* < 0.0001).

#### Anterior displacement

**Fig. 4:**
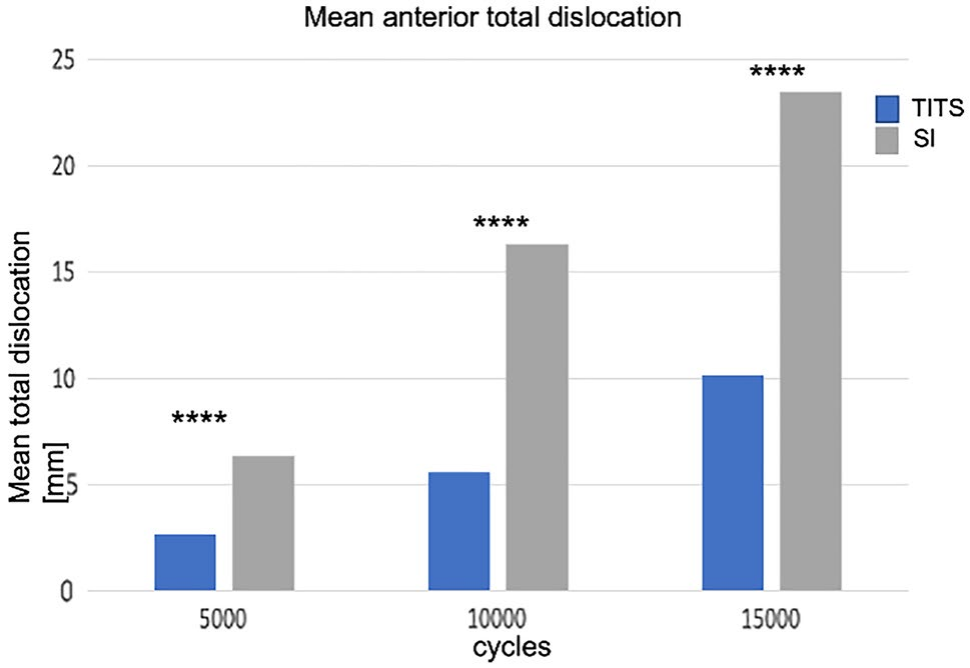
Total dislocation of the anterior pelvic ring. Bar charts show mean values for the groups every 5000 cycles. Significant difference are marked with *p < 0.05

The mean anterior total dislocation was significantly higher in the SI group. The highest total dislocation difference between the groups was seen between 11,000 and 15,000 cycles with 13.3 mm (*p* < 0.0001) The anterior displacement for every 5000 cycles is shown in Fig. 4.

### Radiographic results

**Fig. 5:**
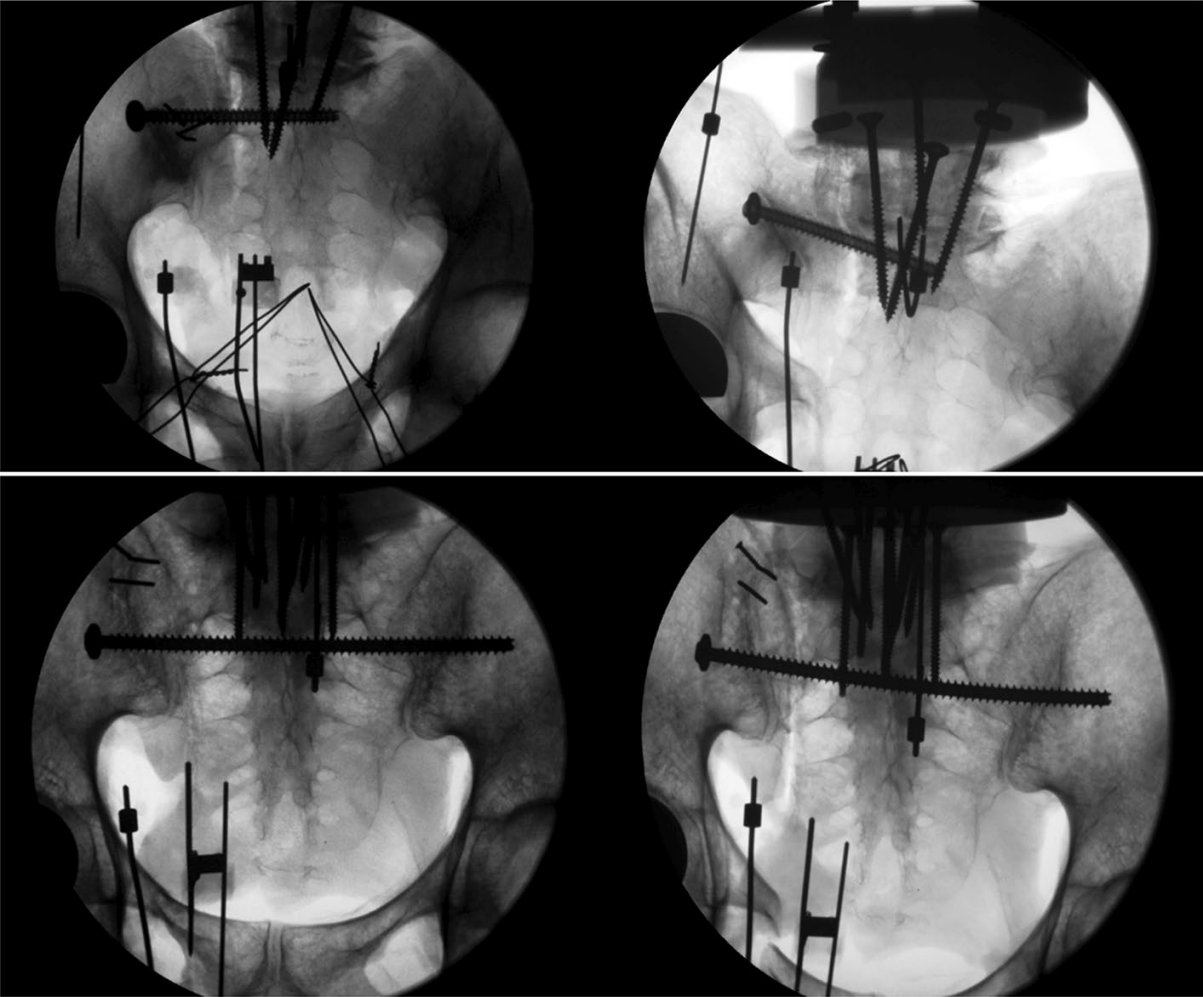
Exemplified anterior–posterior radiographs of a construct instrumented with and SI-Screw (upper) and TITS-Screw (lower), shown at test start (left), and after 15,000 cycles (right)

The SI group showed vertical displacement due to screw penetration through the cancellous bone. In the TITS group radiographic vertical displacement was less and a bending of the screw was seen in all cases (Fig. 5).

## Discussion

This study compared the biomechanical stability of anterior and posterior pelvic ring fractures after osteosynthesis with TITS or SI screws in an osteoporotic human cadaveric model. Axial stiffness as well as implant failure showed no significant differences in the cohorts. This suggests that both surgical procedures are initially adequate to stabilize an osteoporotic pelvic ring fracture. However, initial axial stiffness is not prognostic for durability of osteosynthesis after loading, a criterion of utmost relevance in osteoporotic bone. Complicated healing processes in the sense of screw loosening and screw dislocation with associated loss of fracture fixation, especially with sacroiliac screws, have been documented in the literature [18, 21]. A clear trend towards higher stability of the long screw could be demonstrated. Under biomechanical stability criteria, significantly reduced interfragmentary motion of the pelvis with long screw was shown in all cases. This demonstrates the biomechanical superiority of a TITS screw to achieve a more stable fracture treatment in osteoporotic pelvises.

To provide the best possible treatment for osteoporotic pelvic ring fractures, it was first necessary to determine the fracture morphology of these injuries. Scheyerer et al. were able to demonstrate a posterior pelvic ring lesion in elderly patients with pubic ramus fractures in over 96.8% [22]. In presenting their classification, Rommens and Hofmann showed that the primary injury patterns in osteoporotic pelvises also showed anterior and posterior involvement of the annular structure. In the 243 pelvises studied, the most common fracture morphology was FFP-IIb and -Iic [18]. Accordingly, a fracture according to FFP-IIc was considered for this test setup to provide the highest possible clinical relevance.

There is currently no scientifically based guideline for osteoporotic pelvic ring fracture treatment, even though there is a broad consensus in the community on a surgical approach. In a retrospective study, Walker et al. demonstrated the advantages of surgical management over conservatively managed osteoporotic pelvic fractures. Thus, patients in the surgically managed cohort benefited from a reduction in pain, better mobilization, as well as discharge to home, as opposed to a nursing facility [7]. Similar results were obtained by Maier et al. with a poor outcome of conservatively treated osteoporotic pelvic ring fractures [23]. 

Numerous osteosynthesis techniques have been developed and discussed in the past for stabilization of osteoporotic and non-osteoporotic pelvic ring fractures. Nowadays, a variety of osteosynthetic options are available to us: transiliac and other plate osteosynthesis procedures of the posterior and anterior pelvic rings, sacroiliac screws, sacral bars, lumbopelvic fixation, and triangular osteosynthesis as a combination of the different methods [9, 24–28]. If we focus on the osteosynthetic methods performed in this study, Schuetze et al. demonstrated in their clinical study that the SI screws as well as the TITS screws showed a good clinical success in the treatment of osteoporotic posterior pelvic ring fractures. In addition, a lower complication rate was shown in the TITS group [26]. 

With regard to the biomechanical aspects of the various osteosynthesis modalities and fracture types, Peng et al. were able to show in their study that in H- and U-type fractures the common methods such as lumbopelvic support and bilateral triangular stabilization achieve a stable result. However, TITS screw osteosynthesis was shown to be the most stable in all aspects studied [29]. Van Zwienen et al. as well as Yinger et al. found, in their biomechanical study, that the use of two instead of one short sacroiliac screw increases the rotational stiffness and improves the load of failure [15, 16]. In a similar experimental setup as in the present study, Tabaie et al. demonstrated that additional compression by a locked transsacral screw showed significantly increased stability in Type-C-Fractures compared to 2 sacroiliac screws [17]. On the contrary, Salari et al. found no significant difference in their biomechanical study comparing long iliosacral screws with transsacral screws in type C pelvic ring fractures [12]. Considering the simultaneous stabilization of the anterior pelvic ring in type C fractures, Lodde et al. in their biomechanical study was able to achieve higher stability using a retrograde pubis screw and a sacroiliac screw [30]. In their biomechanical study, Kußmaul et al. came to similar results. Here, the additional stabilization of the anterior pelvic ring was more stable, but statistical significance could not be achieved [31]. However, in both studies, a TITS screw was not considered. The literature indicates that the additional fixation of the anterior pelvic ring shows an advantage in terms of stability, especially in FFP II fractures [32, 33]. When plate osteosynthesis is considered, double plate osteosynthesis of the anterior pelvic ring showed an advantage regarding the screw loosening rate compared to single plate osteosynthesis [34]. 

To improve screw fixation, Suero et al. showed in their biomechanical study better results for augmented sacroiliac screws and TITS screw fixation compared to non-augmented short screws [11]. However, it should be noted that augmentation of the screws bears the risk of cement leakage as well as TITS osteosynthesis shows a higher risk of malposition and neurological damage [35, 36].

This study has several limitations. As in most biomechanical studies, the small group size does not allow definitive conclusions about any statistical significances. Moreover, it is in the nature of biomechanical studies that they can be translated into reality theoretically, but only to a limited extent practically. Degenerative pre-existing conditions could alter the outcome outside the test setup. Additional pulling and compressive forces from surrounding muscles allow only limited clinical relevance. Fixation of the anterior pelvic ring may provide better stability. In further biomechanical studies, the additional stabilization of the anterior pelvic ring in combination with a transiliacal–transsacral screw should be investigated.

## Conclusion

In this study, we could show that the use of transiliacal–transsacral screws in FFP-IIc fractures in osteoporotic pelvic fractures is biomechanically advantageous in almost all aspects investigated, such as flexion, vertical dislocation and gap angle. In addition, a smaller anterior dislocation has been shown. For clinical practice, we also recommend to consider a minimally invasive TITS screw in osteoporotic FFP IIc fractures. If necessary, osteosynthesis of the anterior pelvic ring should be considered in case of large fragment dislocation.

## Data Availability

The authors confirm that the data supporting the findings of this study are available within the article and its supplementary materials.
